# Prognostic and therapeutic implications of iron-related cell death pathways in acute myeloid leukemia

**DOI:** 10.3389/fonc.2023.1222098

**Published:** 2023-09-05

**Authors:** Tongyu Li, Tongtong Lin, Jiahao Zhu, Miao Zhou, Shufang Fan, Hao Zhou, Qitian Mu, Lixia Sheng, Guifang Ouyang

**Affiliations:** ^1^ Department of Hematology, The First Affiliated Hospital of Ningbo University, Ningbo, Zhejiang, China; ^2^ Ningbo Clinical Research Center for Hematologic Malignancies, The First Affiliated Hospital of Ningbo University, Ningbo, Zhejiang, China; ^3^ Department of Pharmacy, Tsinghua University, Beijing, China; ^4^ Cixi Biomedical Research Institute, Wenzhou Medical University, Ningbo, Zhejiang, China; ^5^ Stem Cell Transplantation Laboratory, The First Affiliated Hospital of Ningbo University, Ningbo, Zhejiang, China

**Keywords:** acute myeloid leukemia, iron-related cell death, apoptosis, prognostic biomarkers, targeted therapy

## Abstract

Acute myeloid leukemia (AML) is a blood cancer that is diverse in terms of its molecular abnormalities and clinical outcomes. Iron homeostasis and cell death pathways play crucial roles in cancer pathogenesis, including AML. The objective of this study was to examine the clinical significance of genes involved in iron-related cell death and apoptotic pathways in AML, with the intention of providing insights that could have prognostic implications and facilitate the development of targeted therapeutic interventions. Gene expression profiles, clinical information, and molecular alterations were integrated from multiple datasets, including TCGA-LAML and GSE71014. Our analysis identified specific molecular subtypes of acute myeloid leukemia (AML) displaying varying outcomes, patterns of immune cell infiltration, and profiles of drug sensitivity for targeted therapies based on the expression of genes involved in iron-related apoptotic and cell death pathways. We further developed a risk model based on four genes, which demonstrated promising prognostic value in both the training and validation cohorts, indicating the potential of this model for clinical decision-making and risk stratification in AML. Subsequently, Western blot analysis showed that the expression levels of C-Myc and CyclinD1 were significantly reduced after CD4 expression levels were knocked down. The findings underscore the potential of iron-related cell death pathways as prognostic biomarkers and therapeutic targets in AML, paving the way for further research aimed at understanding the molecular mechanisms underlying the correlation between iron balance, apoptosis regulation, and immune modulation in the bone marrow microenvironment.

## 1 Introduction

Acute myeloid leukemia (AML) is a diverse malignancy of the blood that disrupts normal hemopoietic processes and promotes the excessive proliferation of myeloid cells within the bone marrow (1, 2). About 80% of all cases of AML are seen in aged group (3–5). The overall 5-year mortality survival proportion for people with AML is about 24% despite substantial breakthroughs in our knowledge of AML etiology and the introduction of targeted medicines. Because of the wide range of symptoms and reactions to therapy that patients with AML may experience, it is imperative that innovative prognostic biomarkers and therapeutic techniques be developed to better serve this patient population.

Numerous biological functions rely on iron, including DNA synthesis, energy metabolism, and respiration (6, 7). However, increased iron levels may provoke oxidative stress and cell damage by aiding in the creation of reactive oxygen species (ROS) (8). Iron balance, which is the careful regulation of iron availability and utilization in cells, is precisely controlled, and dysregulation of this balance has been associated with the initiation and advancement of various cancers, such as AML (9, 10). Investigation is underway into the viability of ferroptosis and other mechanisms of iron-related cell death as promising therapeutic avenues for cancer treatment (11, 12). The increase of lipid peroxides distinguishes ferroptosis, which is triggered by iron overload, from other forms of cell death such as apoptosis, necrosis, and autophagy (13). The therapeutic benefits of targeting ferroptosis pathways in AML remain to be fully investigated, despite encouraging findings in preclinical research for a variety of malignancies.

Cancer development, metastasis, and resistance to treatment are all tied closely to the immune microenvironment (14, 15). Cancer cells, immune cells, fibroblasts, endothelial cells, and other cell types, as well as extracellular matrix proteins and signaling chemicals, make up this complex. Relapse and medication resistance are factors in AML because the bone marrow microenvironment offers a favorable habitat for leukemia-initiating cells (16, 17). The incursion of immune cells into the bone marrow microenvironment (BME) has been deemed a vital feature affecting cancer advancement and patient prognostication (18). Programmed cell death receptor-1 (PD-1) and its natural ligand programmed cell death ligand-1 (PD-L1) are two examples of immune checkpoint molecules that have been the focus of immunotherapy in recent years, which have produced durable responses in a diverse set of malignancies (19, 20). However, immune checkpoint inhibitors have had little success in treating AML, underlining the need for a deeper insight of the intricate relationship between leukemia cells and the immune system in order to create more potent immunotherapeutic approaches.

Data from high-throughput sequencing projects and computational biology are now often employed in medical studies (21–23). In an effort to comprehend the origins of disease progression, Wang and colleagues leveraged computational biology methodologies, including WGCNA, to identify biomarkers across multiple cancers (24, 25). This investigation endeavors to explore the potential prognostic implication of iron-associated cell death and apoptotic genes in AML and scrutinize their links with the immune status of the BME. By analyzing transcriptomic data from several publicly available AML datasets, we identified differentially expressed iron-related cell death and apoptosis genes associated with patient prognosis. We subsequently utilized unsupervised clustering to separate AML patients into distinct molecular subpopulations based on expression patterns of these genes, and evaluated their connections with clinical response, immune cell infiltration, and drug susceptibility.

Moreover, we probed the plausible interplay between iron-mediated cell death pathways and immune regulation by scrutinizing the expression, methylation, amplification, and deletion patterns of immune regulation-associated genes in the unique AML subtypes. Finally, we established and validated overall clinical outcome risk signature according to the expression of vital differentially expressed genes, to forecast patient survival in AML.

As a result, our discoveries offer novel perspectives on the involvement of iron-mediated cell death pathways in the development of AML and their correlation with the immune - intricacies of the bone marrow microenvironment. Through the delineation of the molecular categories of AML patients based on gene expression related to iron-mediated cell death and apoptosis, we highlight the prognostic potential of these genes and their role in shaping the immune contexture within the BME. Furthermore, our study highlights the complex interplay between iron homeostasis, cell death regulation, and immune regulation, which may have important implications for the development of novel therapeutic strategies in AML.

## 2 Methods

### 2.1 Data collection

Three information repositories were collected for this research. Dataset 1 comprised of TCGA combined with GTEx data (malignant = 173, Normal = 70) from the UCSC Xena website (xenabrowser.net) (26). Dataset 2 included TCGA-LAML data (https://portal.gdc.cancer.gov; n=126, eliminating patients who did not survive for more than 30 days.) obtained using the TCGAbiolinks package, with clinical information sourced from TCGA-CDR- **Supplemental Table S1.xlsx**. Dataset 3 consisted of GSE71014 data (https://www.ncbi.nlm.nih.gov/geo/; n=104, excluding patients with survival times less than 30 days) (27).

The gene set for this study focused on iron-related death and apoptosis genes. Mutation data were downloaded using the TCGAbiolinks package, while CNV and methylation data were obtained from the UCSC Xena website. Unless otherwise specified, all analyses were conducted using the TCGA-LAML dataset, survival-related analyses were performed after excluding patients who had a survival time of fewer than 30 days.

### 2.2 Determination and analysis of ferroptosis and apoptosis protein-coding genes in AML

First, the limma package was used for differential analysis of AML expression data (log2 (TPM+0.01)) from TCGA combined with GTEx obtained from the UCSC Xena website. Next, the 8,542 differentially expressed genes were intersected with 660 iron death and apoptosis gene sets, resulting in 268 differentially expressed iron death or apoptosis genes. The analysis of the mutation patterns of the 268 genes was conducted using the maftools software package which provides a suite of tools for the comprehensive visualization and statistical exploration of somatic mutation data, and their CNV frequencies were determined. Ultimately, the Kaplan-Meier survival analysis was conducted to evaluate the prognostic implications of the 268 dysregulated genes associated with iron-mediated cell death or apoptosis, identifying 69 prognosis-related key genes (p<0.05), consisting of 11 iron death genes and 58 apoptosis genes. The was used to construct a PPI network, which was visualized using Cytoscape software (28).

### 2.3 Cluster analysis and pathway enrichment

Using the ConsensusClusterPlus package, the 69 prognosis-related genes identified in the TCGA-LAML dataset were subjected to consensus clustering. The optimal number of clusters was determined by analyzing cophenetic, dispersion, and silhouette data. The GSVA package was employed to calculate iron death and apoptosis scores. Differential gene expression between the two clusters and the GSVA-derived 50 hallmark pathway scores were also analyzed. Employing the limma R package, a differential analysis between the two distinct molecular clusters was conducted. The aim of this analysis was to evaluate the gene expression differences between Cluster2 (n=65) and Cluster1 (n=61), and to gain critical insight into the gene expression variations underlying the biological heterogeneity observed in AML patients. By leveraging this analysis, key genes driving the variability between clusters can be identified, paving the way for better prognostic and therapeutic outcomes for patients with AML. The clusterProfiler R package was utilized for the elucidation of KEGG and GO enrichment analysis of the differentially expressed genes, which enabled the identification of the functional pathways associated with these key genes. This analysis has enabled researchers to shed light on the potential biological significance of these genes in the pathogenesis of AML and their probable involvement in the manifestation of the observed heterogeneity within the cancer biology.

### 2.4 Drug correlation evaluation

The oncoPredits package was utilized to conduct drug sensitivity analysis, which allowed for the measurement of the sensitivity of different molecular subtypes to various drugs. Using the limma package, differential analysis of drug sensitivity results was performed, with the goal of identifying drugs that were differentially responsive in the distinct AML subtypes. Subsequently, the top eight drugs exhibiting the largest differences in upregulation and downregulation between the subtypes were selected for further analysis. The selection of these drugs was based on their potential therapeutic significance in the context of AML. Finally, to summarize the differences in drug response between the two clusters, boxplots were used to compare the selected drugs. This analysis provided novel insights into the potential therapeutic targets of AML subtypes, which is crucial in the development of effective personalized treatment plans.

### 2.5 ESTIMATE score and bone marrow microenvironment correlation analysishe

ESTIMATE algorithm was applied to calculate the immune and stromal scores for patients belonging to clusters cluster1 and cluster2. The ESTIMATE algorithm, which is based on gene expression signatures, can evaluate the degree of immune infiltration and stromal activation within malignant samples, and can be useful in deciphering the role of the bone marrow microenvironment in AML pathogenesis. Thus, utilizing the ESTIMATE scores, potential differences in the immune context of the different clusters can be assessed, enabling a more comprehensive understanding of the biological heterogeneity underlying AML. Immune checkpoint gene expression, methylation, amplification frequency, and deletion frequency were also analyzed using the IOBR package and various other methods, such as cibersort, EPIC, MCPCounter, and quantiseq.

### 2.6 LASSO-cox regression analysis and risk model development

A univariate Cox regression analysis was conducted on 342 differentially expressed genes (cluster 2 versus cluster 1) using both the TCGA-LAML and GSE71014 datasets. Analysis was performed to assess the prognostic value of these genes and their association with overall patient survival. Genes having a p-value<0.05 were considered significant. Utilizing this analysis, potential prognostic factors were identified, that can be integrated as part of a personalized treatment protocol for improved disease outcome in AML patients. Applying the LASSO-Cox regression analysis with a 10-fold cross-validation, we evaluated 42 key differentially expressed genes identified during the univariate Cox regression analysis to identify genes with optimal prognostic value. The LASSO-Cox regression approach is a robust method for feature selection, which allows for the identification of the most informative genes in relation to patient survival. The 10-fold cross-validation procedure works to prevent overfitting and helps to identify models that can be suitably generalizable. Consequently, by utilizing this analysis approach, our results allow for an accurate prediction of patient outcomes in AML and are pertinent in developing effective, patient-specific treatment strategies. The risk score was calculated by multiplying the expression value of each gene with its corresponding coefficient, as shown below:


(1) $$\text{Riskscore}=\overset{\text{n}}{\underset{\text{i}=1}{\sum }}\left[expression\;value\;of\;gene_{i}*\beta_{i}\right]$$

The variable “n” represents the number of genes included in the signature, and the variable “β” denotes the coefficient assigned to each gene obtained from LASSO regression.

### 2.7 Cell culture

KG-1α and OCI-AML2 cells were cultured in RPMI-1640 media supplemented with 10% fetal bovine serum (FBS) under optimal conditions of 37 degrees Celsius in a humidified 5% CO_2_ atmosphere. In accordance with standard practices for cell culture, the cells were split every two to three days to ensure continuous logarithmic growth. These culture conditions offer an optimal environment and nutrient supply to support the growth and maintenance of these cell lines in a manner that is consistent with previous culture methods.

### 2.8 siRNA transfection

Lipofectamine 3000 (Invitrogen) was used to transfect CD4 siRNA and control siRNA into KG-1α and OCI-AML2 cells. After 6 hours of incubation with the siRNA complexes, the cells were given a fresh supply of media. The cells were collected for examination 48 hours after transfection. The siRNA sequence is as follows: Negative control: Sense: 5′-UUCUCCGAACGUGUCACGUTT-3′, Antisense: 5′-ACGUGACACGUUCGGAGAATT-3′; si-CD4: Sense: 5′- CCCUGAUCAUCAAGAAUCUTT-3′, Antisense: 5′- AGAUUCUUGAUGAUCAGGGTT-3′.

### 2.9 Western blotting

Total cellular proteins were extracted from transfected cells and the protein concentration was measured using a BCA Protein Assay Kit (Pierce). The extracted proteins were then separated by SDS-PAGE and transferred onto PVDF membranes. To ensure equal quantities of each target protein, membranes were probed with primary antibodies against CD4 (Cat No. 67786-1-Ig; Proteintech), C-Myc (Cat No. 67447-1-Ig; Proteintech), Cyclin D1 (Cat No. 60186-1-Ig; Proteintech), and Vinculin (Cat No. 66305-1-Ig; Proteintech). Following incubation with horseradish peroxidase-conjugated secondary antibodies, the ECL Western Blotting Detection Reagents (GE Healthcare) were used to visualize the membranes and generate chemiluminescent signals. The results of the experiment were analyzed using ImageJ (NIH) and densitometry was applied to measure the thickness of protein bands in the processed images. This rigorous analysis approach allowed for the accurate quantification and comparison of protein samples and resulted in the generation of robust, reliable data.

## 3 Results

### 3.1 Identification of differentially expressed iron-related cell death genes

**Figure 1:**
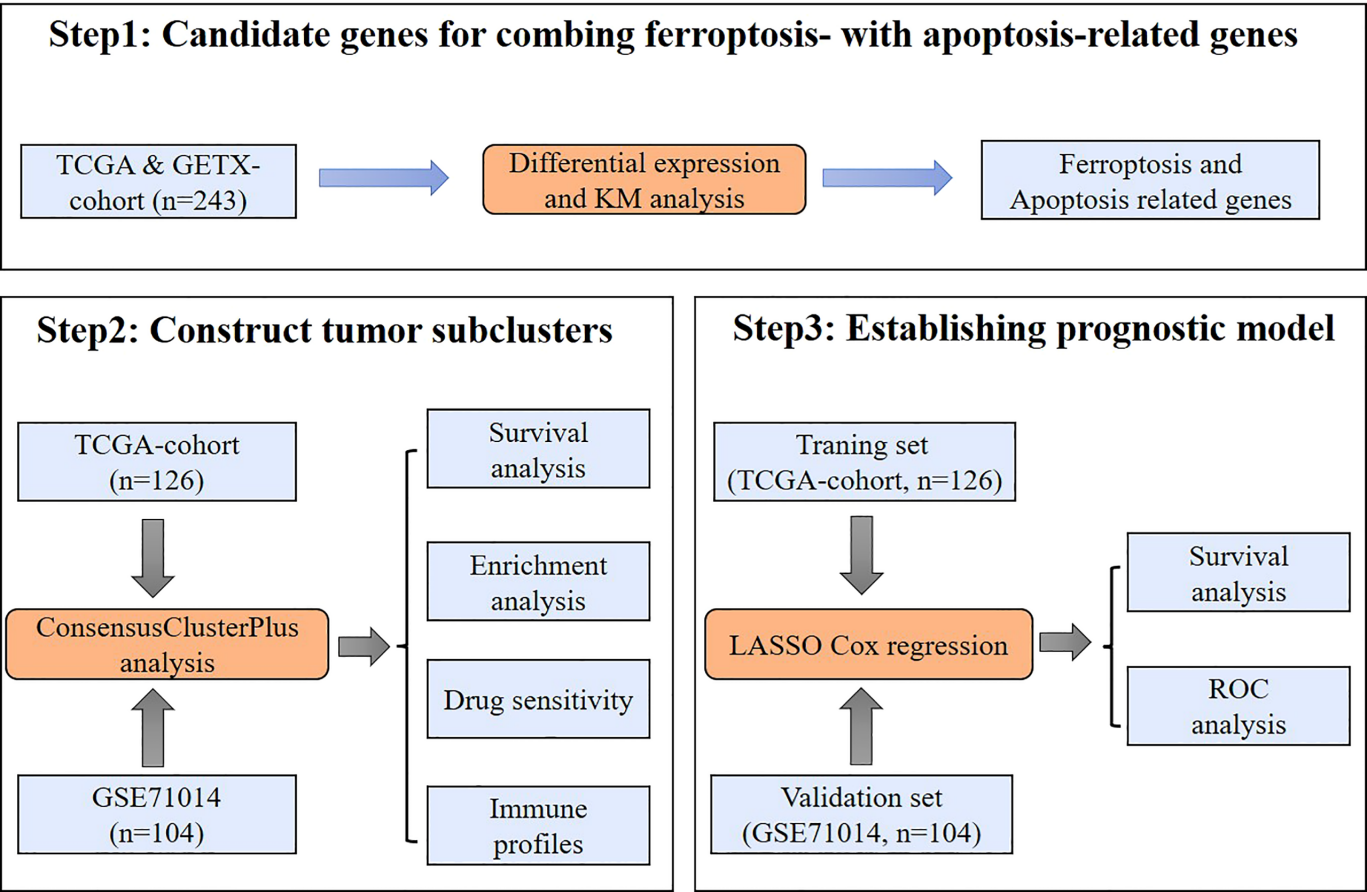
Study workflow. A schematic representation of the data collection, analysis, and validation processes used in this study.

**Figure 2:**
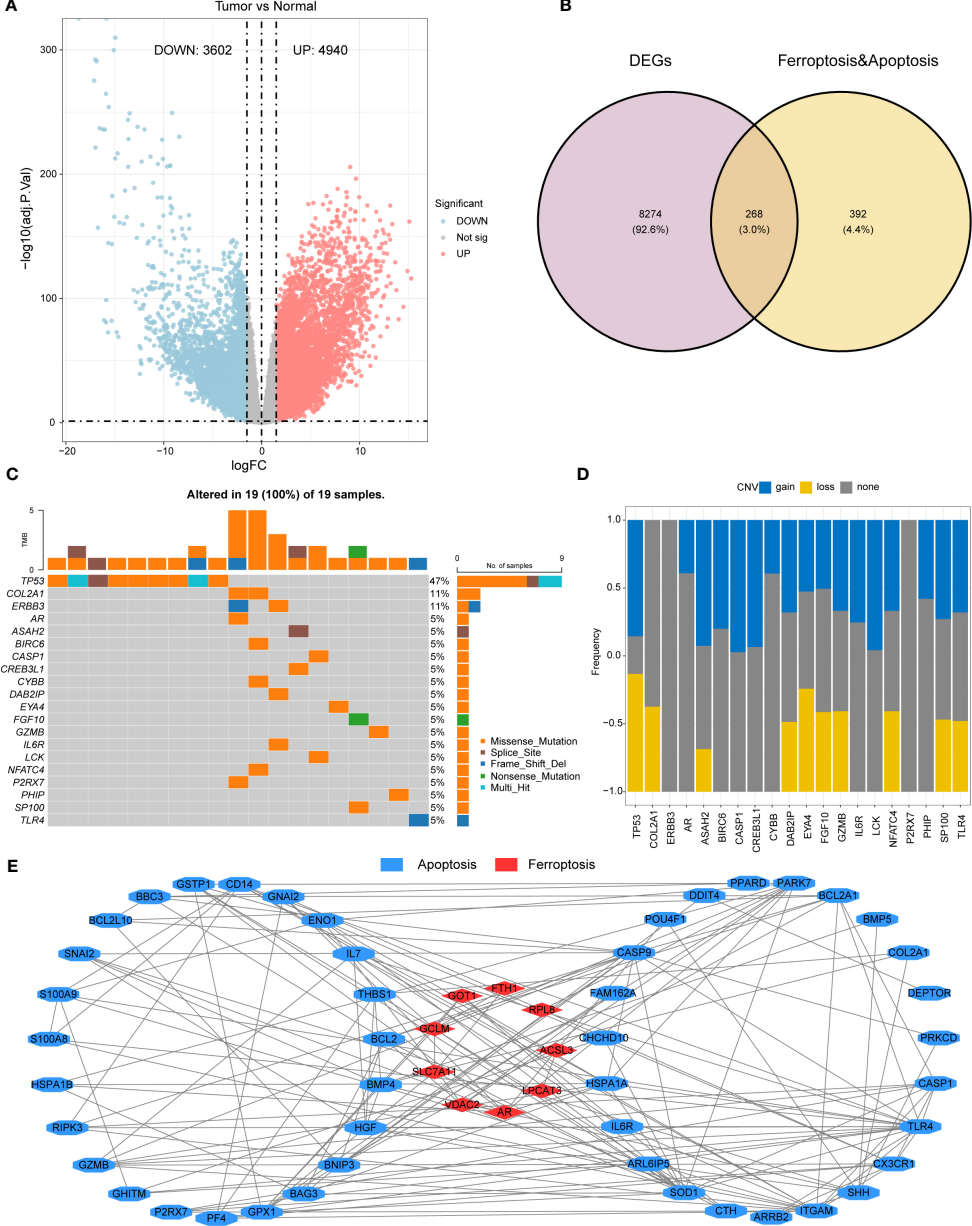
Differential gene expression and mutation analysis of iron-related cell death and apoptosis genes **(A)** Volcano plot demonstrating the differentiallyexpressed genes (DEGs) in the AML malignant samples relative to the normal control samples. **(B)** A Venn diagram providing visualization of theoverlap between DEGs and iron-related apoptosis and cellular death genes. **(C)** A waterfall plot illustrating the top 20 mutated genes out of 268DEGs related to iron-mediated apoptosis and cellular death genes. **(D)** A bar plot showing the CNV (copy number variation) frequencies of the top20 mutated genes. **(E)** A protein-protein interaction network was constructed using the STRING database, and visualized in Cytoscape,demonstrating the 69 prognostic key genes.

**Figure 1** shows the workflow of this study. AML expression data (log2 (TPM+0.01)) from the TCGA combined with GTEx dataset was obtained from the UCSC Xena website. The limma package was utilized to conduct a differential expression analysis. The obtained results were visualized by generating a volcano plot (**Figure 2A**), which depicts the distribution of differentially expressed genes (DEGs) between malignant (n=173) and normal (n=70) samples. Using the criteria of adj.pvalue< 0.05 & |logFC| > 1.5, the analysis identified a total of 4940 upregulated genes and 3602 downregulated genes. These genes were considered to be significantly differentially expressed, with their potential involvement in the pathogenesis of the condition making them promising targets for further investigation. Overall, the generated results provided crucial insights into the molecular mechanisms underlying the disease, paving the way for the development of more effective diagnostic and therapeutic interventions. The intersection of the 8,542 DEGs and the 660 iron-related cell death and apoptosis genes resulted in 268 differentially expressed iron-related cell death or apoptosis genes (**Figure 2B**).

### 3.2 Mutation and copy number variation analysis

Using the maftools package, the mutation status of the 268 differentially expressed iron-related cell death or apoptosis genes was analyzed, and the top 20 genes were displayed in a waterfall plot (**Figure 2C**). Among all samples, the TP53 gene had the highest mutation frequency (47%), followed by COL2A1 and ERBB3 (11%). Among all mutation types, missense mutation was the most common.

A bar plot shows the CNV frequency of the top 20 mutated genes (**Figure 2D**). Most genes had both copy number variation (CNV) gain and CNV loss. Only a few genes, such as COL2A1 and AR, existed in only one of these two conditions.

### 3.3 Prognostic key gene identification and network construction

A Kaplan-Meier survival analysis of the 268 differentially expressed iron-related cell death or apoptosis genes identified 69 prognostic key genes (p< 0.05), including 11 iron-related cell death genes and 58 apoptosis genes. The specific gene list can be found in **Supplementary File 1**. The construction of a protein-protein interaction (PPI) network was carried out through the use of data obtained from the STRING database, which provides a comprehensive resource for exploring known and predicted PPIs (**Figure 2E**). The obtained network was used to basic interaction analysis via a visualization method of Cytoscape software. Utilizing these tools and resources, potential biomarkers and therapeutic targets can be identified, aiding in the development of innovative approaches for the treatment of various human malignancies.

### 3.4 Identify prognostic factors associated with different malignant subtypes

**Figure 3:**
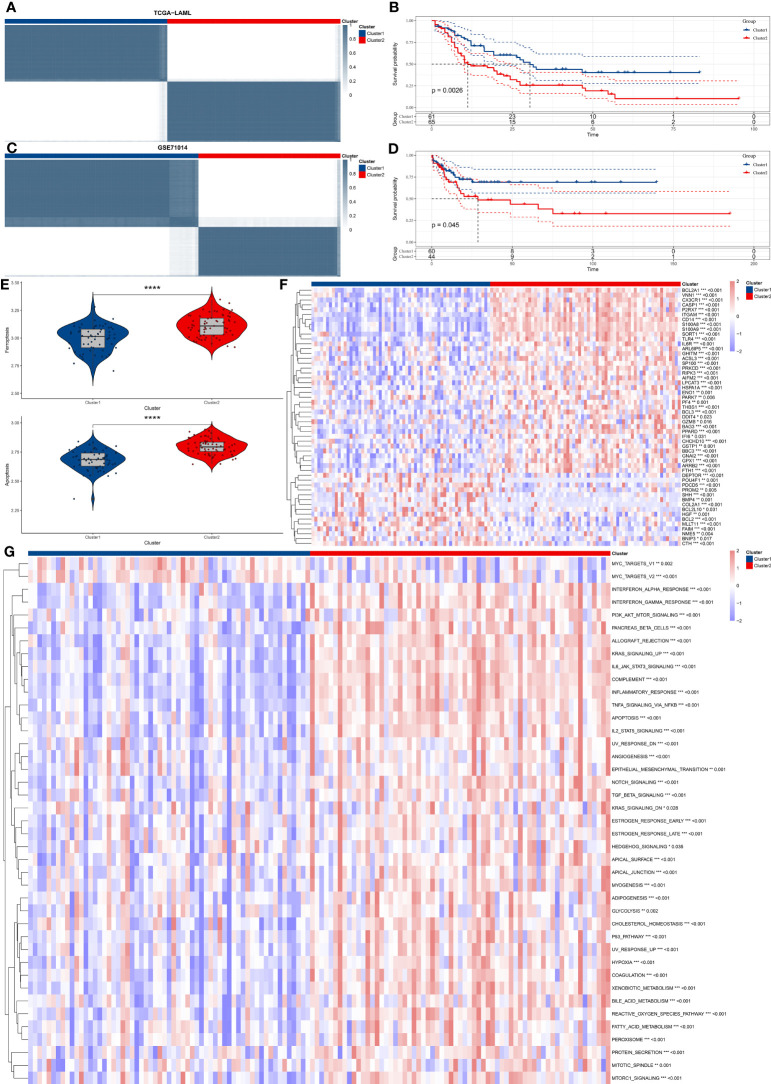
Consensus clustering and functional analysis of AML subtypes. **(A)** The figure shows consensus clustering of AML patients based on the gene expression profile of 69 prognostic key genes. **(B)** Kaplan-Meier survival analysis distinguishes two identified clusters, comparing overall survival between the two groups. **(C, D)**. Validation of clustering findings in the GSE71014 dataset. **(E)** GSVA scores show that the iron-related cellular death and apoptosis pathways significantly vary in the two discovered clusters. **(F)** The Heatmap graphically represents differential gene expression levels of 69 prognostic key genes between the two clusters. **(G)** The Heatmap shows differentially activated HALLMARK pathways between the two identified clusters. "*" represents p<0.05,"**" represents p<0.01,"***" represents p<0.001,"****" represents p<0.0001.

Based on the 69 prognostic key genes identified in **Figure 2E**, the ConsensusClusterPlus package was used to perform consensus clustering on the TCGA-LAML dataset. We used these genes to construct two different expression patterns in an attempt to compare survival outcomes between the different expression patterns. Patients were divided into two clusters (**Figure 3A**). The survival curve indicated that patients in cluster 1 were significantly associated with better prognosis (**Figure 3B**). This finding was validated in the GSE71014 dataset (**Figures 3C, D**). The GSVA scores reveal significant differences in iron-related cell death and apoptosis pathways between the two distinct expression patterns identified above, with these pathways having higher GSVA scores in Cluster 2 (**Figure 3E**). The expression levels of the 69 prognostic key genes in different expression patterns are depicted in **Figure 3F**, showing significant differential expression across both clusters. Finally, the GSVA algorithm was used to compare the enrichment levels of HALLMARK pathways in different expression patterns. The most significantly different pathways are visualized in a heatmap, revealing that pathways such as interferon and inflammatory response are significantly upregulated in Cluster 2 (**Figure 3G**).

### 3.5 Functional enrichment and differential expression analysis of AML clusters

A differential expression analysis between the two distinct molecular clusters (Cluster 2: n=65 and Cluster 1: n=61) was carried out by employing the limma package, a widely used tool for the visualization and analysis of differential gene expression data. Subsequently, this analysis enabled a systematic comparison of gene expression levels between the two clusters, facilitating the identification of the genes that were differentially regulated. The approach enabled a deeper understanding of the molecular basis of AML and provides insights into disease mechanisms that could contribute to the development of more efficient therapies. A volcano plot shows 328 upregulated genes and 14 downregulated genes (**Figure S1A**), with the specific gene list available in **Supplementary File 1**. In order to select differentially expressed genes (DEGs), particular selection criteria were implemented, requiring an adjusted p-value of less than 0.05 and an absolute log-fold change of greater than 1.5. Subsequently, these DEGs (a total of 342) underwent functional enrichment analysis, which was carried out using the clusterProfiler package, and the results of this analysis are presented in **Figures S1B, C**.

### 3.6 Drug efficacy evaluation

**Figure 4:**
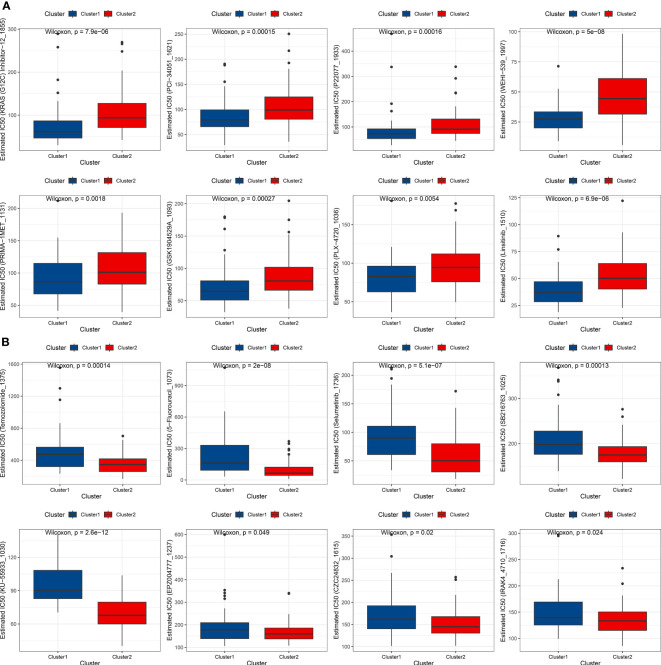
Drug sensitivity analysis of AML subtypes. **(A)** Box plot showing the top 8 most sensitive drugs for cluster 1 patients. **(B)** Box plot displaying the top 8 most sensitive drugs for cluster 2 patients.

To evaluate the efficacy of drugs, the oncoPredits package, which is a powerful tool for predicting drug sensitivity, was employed. This software provides comprehensive analysis of various cancer drugs’ potential effectiveness based on individual patients’ genetic profiles, enabling personalized treatment options and targeted therapeutic approaches to limit side effects. It helps address potential drug resistance and treatment inefficacy, leading to informed decisions and optimal clinical outcomes for patients. Differences in drug sensitivity between the subtypes were analyzed using the limma package, and box plots were created to compare the top 8 most sensitive drugs for each cluster. The top 8 most sensitive drugs for cluster 1 patients were KRAS (G12C) Inhibitor-12_1855, PCI-34051_1621, P22077_1933, WEHI-539_1997, PRIMA-1MET_1131, GSK1904529A_1093, PLX-4720_1036, and insitinib_1510 (**Figure 4A**). The top 8 most sensitive drugs for cluster 2 patients were Temozolomide_1375, 5-Fluorouracil_1073, Selumetinib_1736, SB216763_1025, KU-55933_1030, EPZ004777_1237, CZC24832_1615, and IRAK4_4710_1716 (**Figure 4B**).

### 3.7 Bone marrow microenvironment and immune infiltration analysis

**Figure 5:**
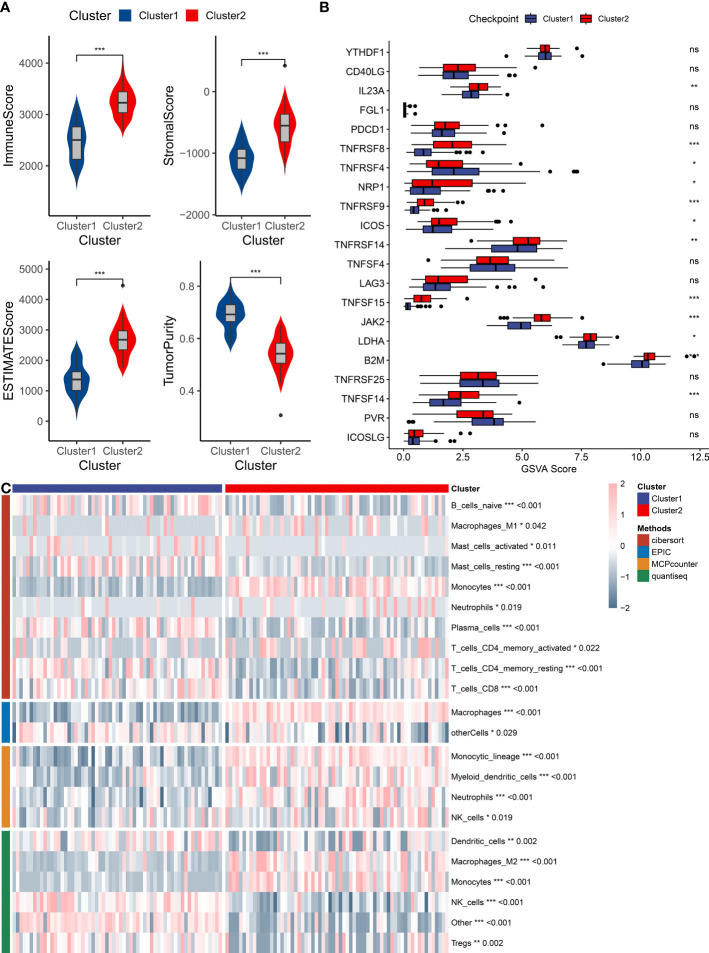
Immune infiltration analysis of AML subtypes. **(A)** A box plot compares immune infiltration scores, stromal levels, ESTIMATE scores, and tumor purity between the two identified clusters. **(B)** A box plot represents differential gene expressions of immune checkpoint genes between the two clusters. **(C)** A heatmap displays immune cell infiltration patterns in the bone marrow microenvironment of the two clusters, as analyzed by CIBERSORT, EPIC, MCPCounter, and QuantiSeq algorithms. "*" represents p<0.05,"**" represents p<0.01,"***" represents p<0.001.

The bone marrow microenvironment was assessed in patients corresponding to clusters 1 and 2 by utilizing the ESTIMATE algorithm via the IOBR package. The obtained results exhibited that patients in cluster 2 had a significantly higher immune score, stromal score, and ESTIMATE score than did patients in cluster 1, while the tumor purity scores were found to be lower in cluster 2 patients (**Figure 5A**). Moreover, the expression of the majority of immune checkpoint genes was found to be significantly upregulated in patients of cluster 2 (**Figure 5B**). In order to further investigate immune infiltration patterns, immune cell infiltration was evaluated, utilizing the IOBR package, and employing several methods such as cibersort, EPIC, MCPCounter, and quantiseq. Heatmaps were employed to visualize distinctive patterns of immune cell infiltration in the bone marrow microenvironment of cluster 1 and cluster 2 patients, with a focus on cell lineages that exhibited statistically significant differences (depicted in **Figure 5C**). This comprehensive analysis of immune infiltration and microenvironmental variations established significant differential characteristics in different expression patterns, which could provide insights into the underlying mechanisms of tumor initiation, growth, and differentiation.

### 3.8 Analysis of immune regulatory genes

**Figure 6:**
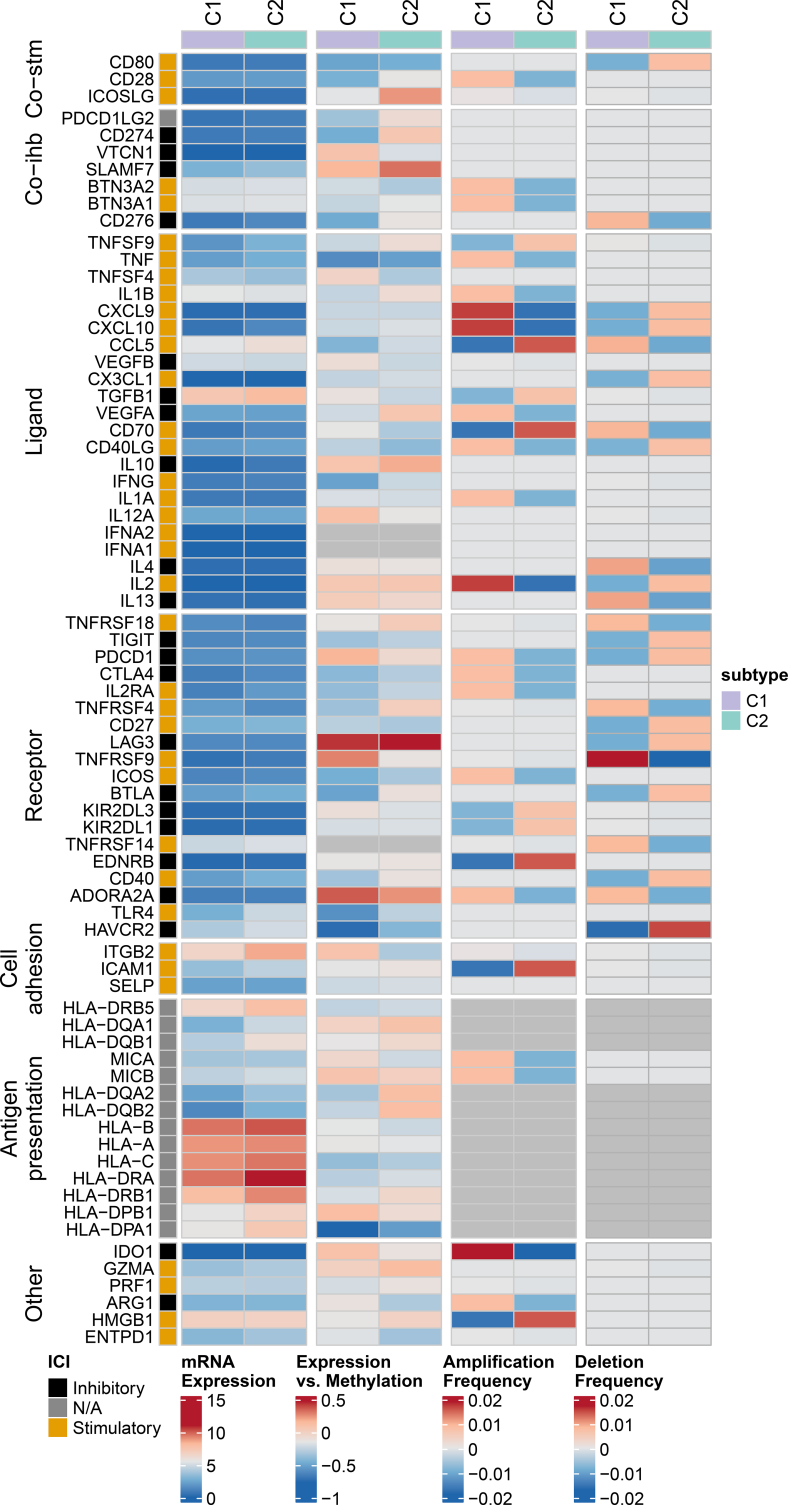
Multi-omics analysis of immune regulatory genes in AML subtypes. Heatmap showing expression levels, methylation levels, amplification frequencies, and deletion frequencies of immune regulatory genes in different groups patients.

Heatmaps were used to present the expression levels, methylation levels, amplification frequencies, and deletion frequencies of immune regulatory genes in different expression patterns (**Figure 6**). There are differential expression levels of antigen presentation-related genes between the two clusters. The two clusters also exhibit differences in the expression/methylation of various immune processes. Moreover, significant variations can be observed in the amplification and deletion frequencies of multiple immune processes between the two clusters. More details on this analysis can be found at https://linkinghub.elsevier.com/retrieve/pii/S1074761318301213.

### 3.9 Construction of a risk prediction model using differential gene expression data

Using the TCGA-LAML and GSE71014 datasets, 342 differentially expressed genes (cluster 2 vs. cluster 1) were subjected to univariate Cox regression analysis, Selecting genes with p<0.05, and intersecting the results to obtain 42 key prognostic differential genes (**Supplementary File 1**). In the TCGA-LAML dataset, LASSO-Cox regression and ten-fold cross-validation techniques were utilized to screen the 42 differential genes obtained from the above-mentioned results, ultimately resulting in a 4-gene risk model (**Figures S2A, B**). The scoring system for predicting risk was derived from the following formula: riskScore = 0.047473*VDR + 0.055492*CD4 + 0.093657*LST1 - 0.17468*SIX3.

**Figure 7:**
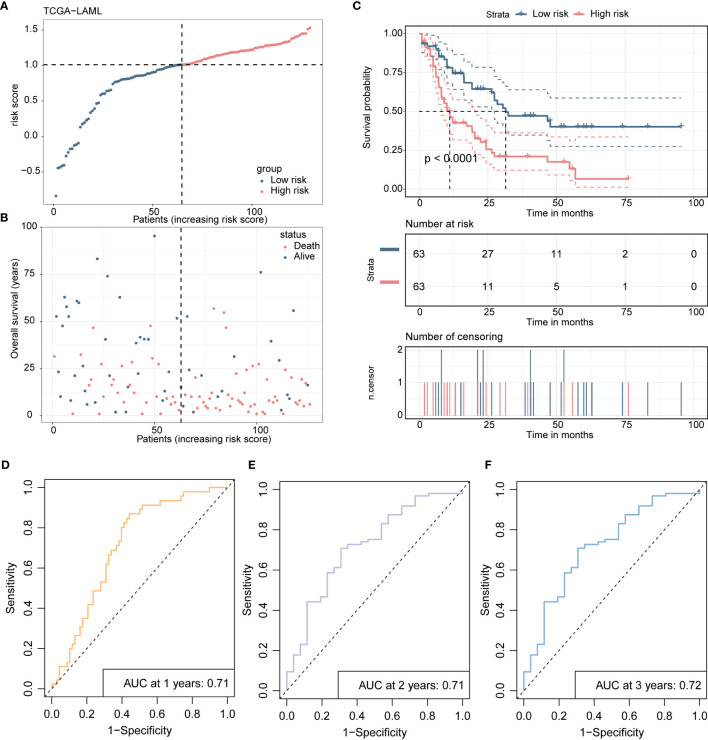
Presents the development and validation procedure of a 4-gene risk model in the TCGA-LAML training cohort, as explained below: **(A)** The distribution of risk scores and survival status in the training cohort are visualized using a dot plot. **(B)** The scatter plot showing the relationship between patient survival probability and risk scores is plotted. **(C)** Kaplan-Meier survival analysis is conducted, comparing overall survival between high- and low-risk groups. **(D-F)**. Receiver Operating Characteristic (ROC) curves indicate the prognostic performance of the risk model at 1, 2, and 3 years.

**Figure 8:**
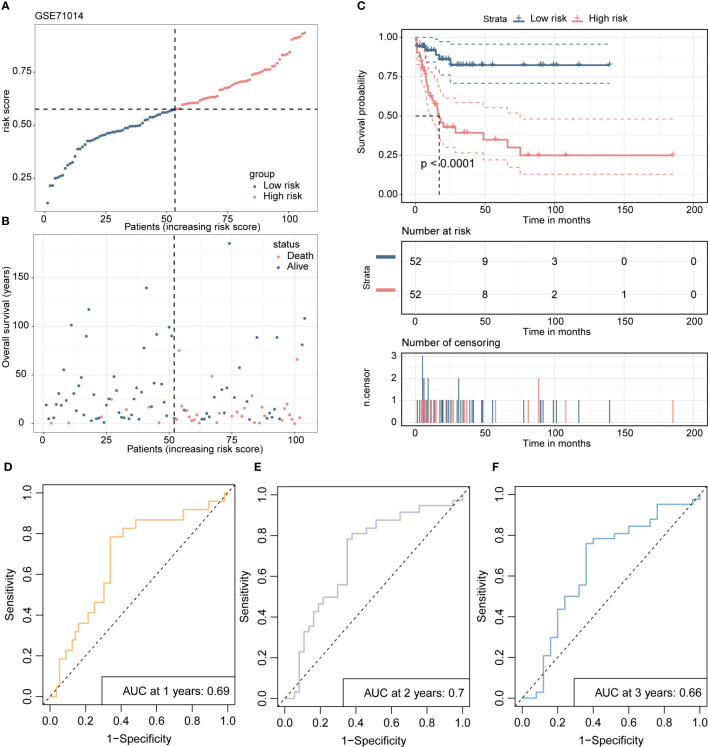
The validation process of the 4-gene risk model is presented using the GSE71014 validation cohort. **(A)** The distribution of risk scores and survival status in the validation cohort is displayed through a dot plot. **(B)** A scatter plot is presented to show the correlation between patient survival probability and risk scores. **(C)** Kaplan-Meier survival analysis is conducted to compare overall survival between high- and low-risk groups. **(D-F)**. The ROC curves demonstrate the prognostic performance of the 4-gene risk model for 1, 2, and 3 years in terms of distinguishing between high- and low-risk groups.

Scatterplots and dot plots revealed that the probability of death also increased for the patients in the high-risk group (**Figures 7A, B**). Survival curves for the two groups demonstrated statistically significant differences (**Figure 7C**), and ROC curves displayed the AUC values of the risk score for predicting different year survival probability, with all values greater than 0.7 (**Figures 7D-F**). Similar results were obtained in the GSE71014 validation dataset (**Figures 8A-F**).

### 3.10 Effects of signature risk gene CD4 in cell cycle in KG-1α and OCI-AML2 cell lines

**Figure 9:**
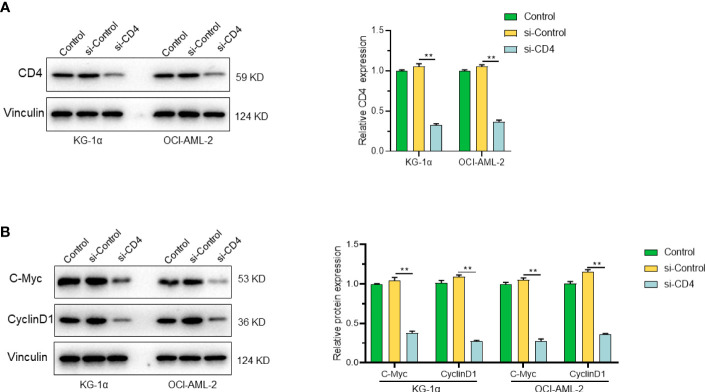
CD4 and its role in cell proliferation. **(A)** Expression of CD4 protein in KG-1α and OCI-AML2 cells after CD4 silencing. **(B)** The expression levels of C-Myc and Cyclin D1 were significantly decreased after CD4 knockdown in KG-1α and OCI-AML2 cell lines. "**" represents p<0.01.

After constructing prognostic gene models in leukemia, we found that CD4, VDR and LST1 were the most significant prognostic risk genes. After reviewing the literature and cell experiments, we found that CD4 may have a significant correlation with the cell cycle of leukemia. The results showed that the expression levels of cyclin C-Myc and Cyclin D1 were significantly decreased after CD4 knockdown in KG-1α and OCI-AML2 cell lines (**Figures 9A, B**). The corresponding statistical data were also shown.

## 4 Discussion

In this current study, we embarked on a comprehensive investigation to determine the influence of iron-related genes on the initiation, progression, and prognosis of AML. By utilizing sophisticated techniques, we identified distinct molecular subtypes that exhibited variations in immune cell infiltration patterns, prognosis, and drug sensitivity profiles - highlighting the potential of iron-related genes as prognostic biomarkers and therapeutic possibilities for AML. Our findings from this study increase our overall comprehension of the intricate interactions between iron metabolism, regulation of cell death, and immune modulation within the microenvironment.

Our results align with previous studies emphasizing the important role of iron metabolism in the initiation and progression of cancer. These include studies demonstrating the participation of iron metabolism in the regulation of tumor angiogenesis, cellular proliferation, and apoptosis, among other mechanisms. Cancer cell proliferation, angiogenesis, metastasis, and therapeutic resistance have all been linked to abnormal iron homeostasis (29, 30). Specifically, iron has been linked to oxidative stress, DNA damage, genomic instability, and tumor promotion, development, and survival (31, 32). ROS are produced when iron oxidizes, and they may be harmful to healthy cells. Furthermore, it is established that iron can also trigger a form of non-apoptotic cell death known as ferroptosis, which is distinguished by lipid peroxidation and the resultant rupture of cellular membranes (33, 34). Building on the conclusions of earlier investigations, we demonstrate that genes involved in iron-related apoptosis and cell death pathways exhibit potential prognostic and therapeutic significance in the context of AML. By utilizing advanced computational tools to perform an in-depth analysis of the differential gene expression patterns in patients corresponding to distinct molecular subtypes of AML, we were able to identify genes involved in iron homeostasis and associated death pathways that are significantly linked with clinical outcomes.

The transcript levels of genes implicated in iron-related cellular demise and apoptotic mechanisms allowed us to identify unique molecular subtypes that were linked with diverse outcomes, immune cell infiltration patterns, and medication sensitivity profiles. Our findings support the notion that stratifying patients into molecular subtypes has the potential to improve diagnostic precision, allowing for more reliable forecasting of outcomes related to patient response to treatment and disease progression, ultimately culminating in better clinical decision-making (35–37). In our study, we found that patients in cluster 1, characterized by lower expression of iron-related cell death and apoptosis genes, had a better prognosis and were more sensitive to certain targeted therapies. In contrast, patients in cluster 2, with higher expression of these genes, exhibited a poorer prognosis.

One intriguing finding is the correlation between iron-related cell death pathway, which may indicate a connection between iron homeostasis, cell death control, and immunological regulation. Ferroptosis has been recently studied for its impact on tumor immune contexture and its implications for immunotherapy response (38, 39). For instance, damage-associated molecular patterns (DAMPs) released during ferroptosis have been found to stimulate antigen-presenting cells and cytotoxic T cells, therefore eliciting anticancer immune responses (40, 41). However, ferroptosis may also stimulate immunosuppressive processes that aid tumor immune evasion (42, 43), such as MDSC and the activation of immunological checkpoint markers. Together, these lines of evidence suggest that iron-related cell death and apoptosis genes may influence the immunological landscape of AML patients, and our results contribute to this expanding body of research.

In addition to the prognostic implications of iron-related cell death pathways, our study also highlights their potential therapeutic relevance in AML. Our results revealed that there are disparities in the patients’ sensitivity to specific targeted therapies based on molecular subtype, such as KRAS (G12C) Inhibitor-12_1855 and PCI-34051_1621, while patients in cluster 2 were more sensitive to other drugs, including Temozolomide_1375 and 5-Fluorouracil_1073. These findings suggest that iron-related cell death and apoptosis gene expression profiles could be used to guide personalized treatment strategies for AML patients, potentially improving therapeutic outcomes.

Our study further utilized Western blotting analysis to demonstrate that CD4 silencing significantly reduced the levels of CD4 protein and cell proliferation markers in AML cells, highlighting the impact of CD4 protein in these cells. Altogether, the evidence suggests that CD4-associated pathways represent a valuable target for leukemia management, which warrants further investigation and exploration. The expression of CD4 protein in T-cell malignancies aids in diagnosing certain types of T-cell leukemia and determining leukocyte differentiation and classification. Moreover, targeting CD4 protein with monoclonal antibodies has shown promising results in T-cell-directed therapies, treating CD4-positive lymphomas, leukemias, and autoimmune diseases.

It is important to note that our study has some limitations. Despite the significant results obtained in our study, the sample size of our datasets is relatively limited, which may hinder the generalization of our findings. Future studies with more extensive cohorts and diverse demographic groups would be considered necessary to test and verify our results, further understanding the potential of iron-related cell death in AML. Finally, our study mainly focuses on gene expression profiles, and other molecular alterations, such as genetic mutations, epigenetic modifications, and post-translational modifications. Furthermore, it is plausible that additional factors impact the regulation of iron-associated cell death pathways in AML, suggesting that further investigations may uncover additional mechanisms contributing to the disease’s pathogenesis and prognostic outcomes.

Despite these limitations, our study revealed iron-related cell death pathways in AML pathogenesis and highlights their potential as prognostic biomarkers and therapeutic targets. The results of our study provide a defining impetus for continued research aimed at uncovering the molecular drivers of the intricate relationship between iron homeostasis, cell death regulation, and immune regulation in the bone marrow microenvironment. Our research has unveiled new avenues for further exploration, with attention directed towards uncovering the molecular mechanisms behind the complex interrelationships between iron homeostasis, immune cell infiltration, and cell death regulation in the bone marrow microenvironment. This, in turn, could catalyze the development of novel therapeutic approaches centered around iron-mediated cellular death pathways in AML and other hematological malignancies.

Our investigation has demonstrated the prognostic and therapeutic importance of iron-related cell death and apoptosis genes in AML, identifying distinct molecular subtypes characterized by different clinical outcomes, immune cell infiltration features, and drug sensitivity profiles. These findings underscore the intricate and multifaceted nature of iron homeostasis, cell death modulation, and immune regulation in the bone marrow microenvironment and suggest that manipulating iron-related cellular death pathways presents a potential therapeutic avenue for managing AML. Further research studies are warranted to authenticate our findings, elucidate the underlying molecular mechanisms, and appraise the clinical utility of iron-associated cell death pathways-based biomarkers and treatments in AML patients.

## Data availability statement

The original contributions presented in the study are included in the article/**Supplementary Material**. Further inquiries can be directed to the corresponding authors.

## Ethics statement

The studies involving humans were approved by The First Affiliated Hospital of Ningbo University. The studies were conducted in accordance with the local legislation and institutional requirements. Written informed consent for participation was not required from the participants or the participants’ legal guardians/next of kin in accordance with the national legislation and institutional requirements. Ethical approval was not required for the studies on animals in accordance with the local legislation and institutional requirements because only commercially available established cell lines were used.

## Author contributions

All the author confirmed their contribution to this research. All authors contributed to the article and approved the submitted version.

## Supplementary material

Supplementary Figure 1 Differential gene expression analysis and functional enrichment analysis of the two AML clusters. **(A)**. A volcano plot presents the differentially expressed genes (DEGs) between patients in cluster 2 and cluster 1. **(B)**. The bar plot displays the significantly enriched KEGG pathways that were identified by clusterProfiler analysis of 342 DEGs. **(C)**. The bar plot demonstrates the significantly enriched biological process, cellular component, and molecular function GO terms, which were identified by clusterProfiler analysis of 342 DEGs.

Supplementary Figure 2 Selection of prognostic key genes and development of a 4-gene risk model using LASSO-Cox regression analysis. **(A)** LASSO coefficient profiles of the 42 intersecting prognostic key genes identified in the TCGA-LAML and GSE71014 datasets. **(B)** The selection of tuning parameter (λ) in LASSO-Cox regression analysis was conducted using a robust technique of 10-fold cross-validation.
