# Supplementary figures and images for: Prognostic and therapeutic implications of iron-related cell death pathways in acute myeloid leukemia
Source: Front Oncol. 2023 Sep 5;13:1222098. doi: 10.3389/fonc.2023.1222098 (PMC10509477; doi:10.3389/fonc.2023.1222098)

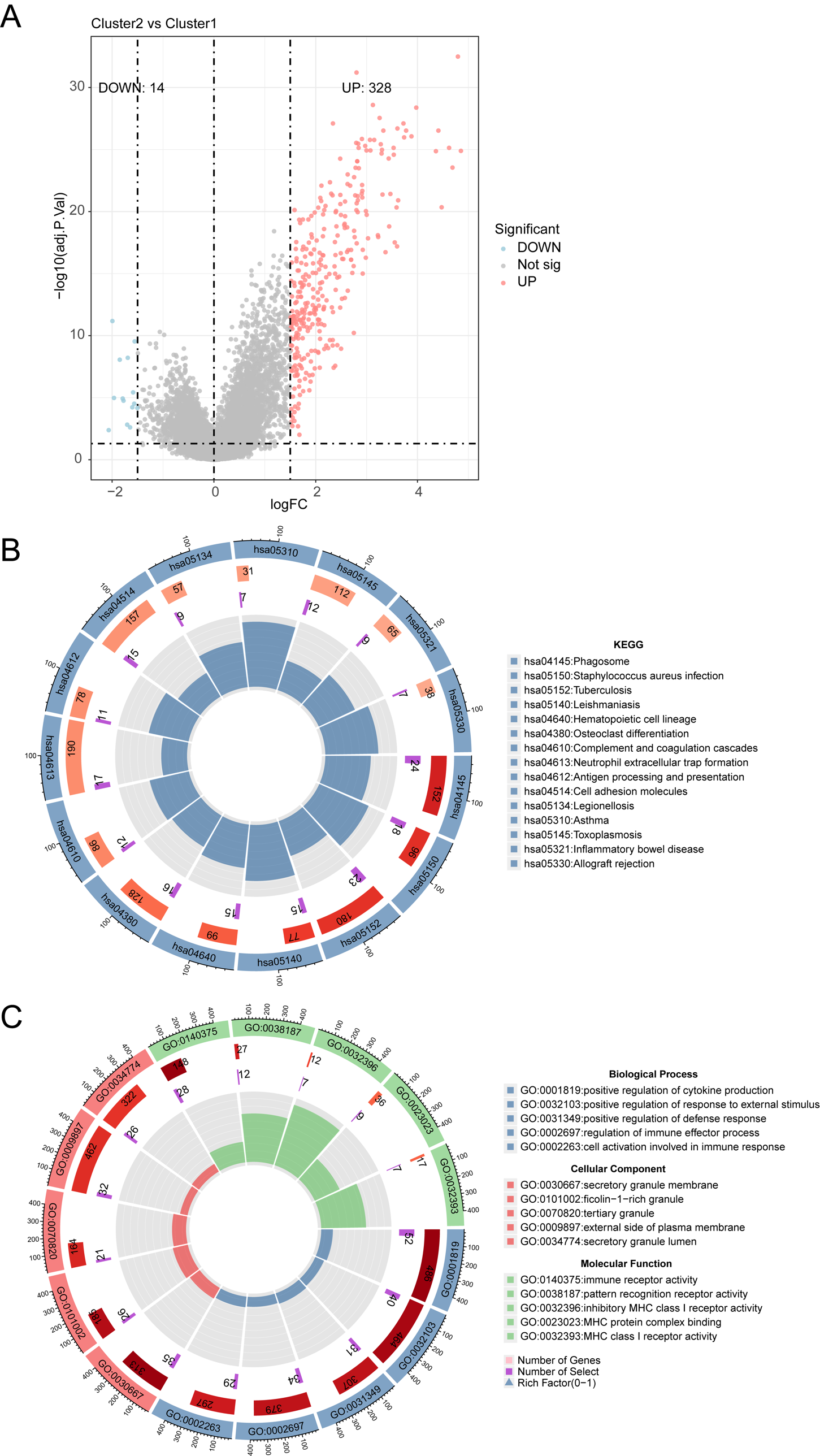

Supplement: Supplementary Figure 1 — Differential gene expression analysis and functional enrichment analysis of the two AML clusters. (A). A volcano plot presents the differentially expressed genes (DEGs) between patients in cluster 2 and cluster 1. (B). The bar plot displays the significantly enriched KEGG pathways that were identified by clusterProfiler analysis of 342 DEGs. (C). The bar plot demonstrates the significantly enriched biological process, cellular component, and molecular function GO terms, which were identified by clusterProfiler analysis of 342 DEGs. [file Image_1.tif]

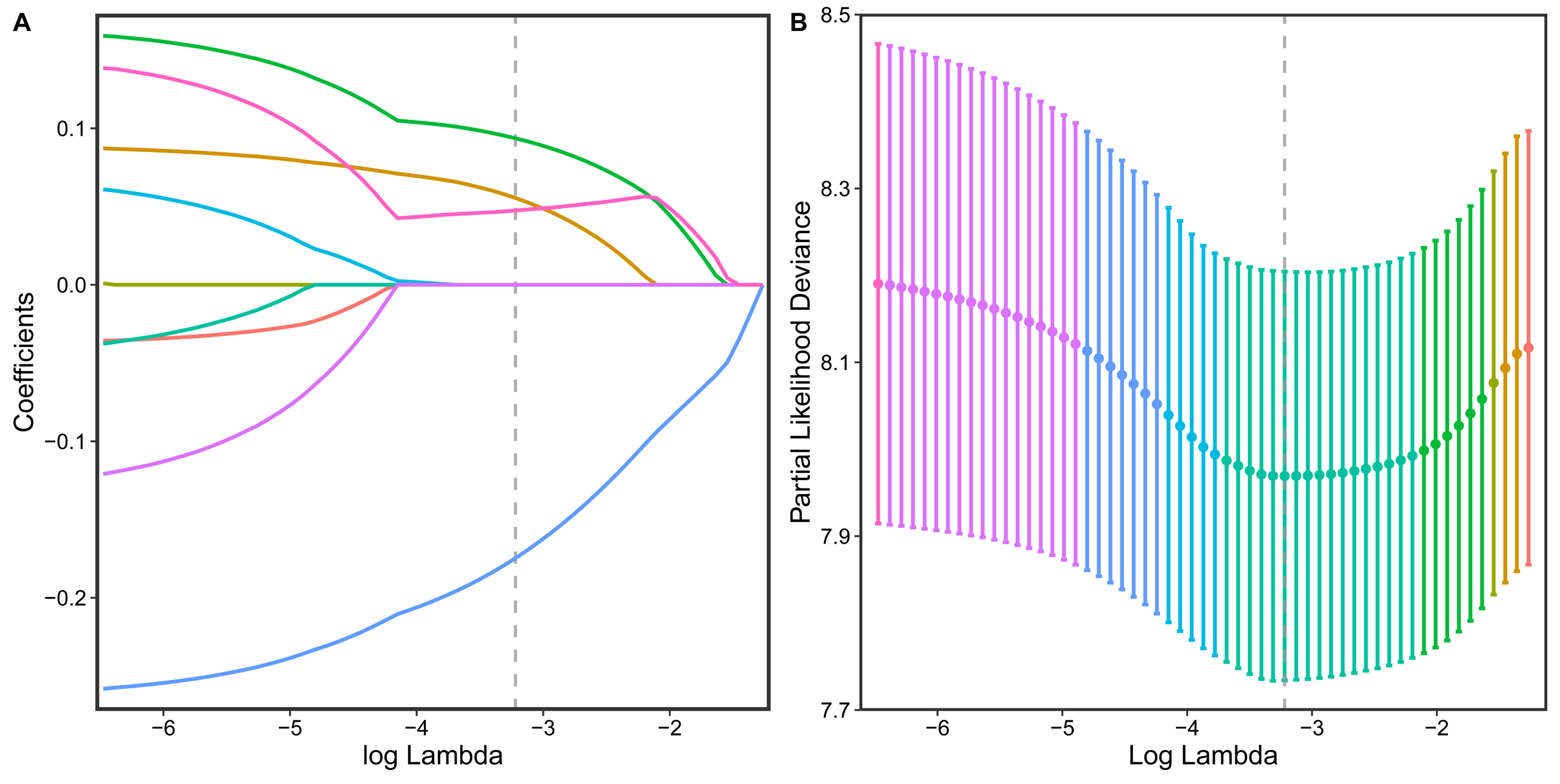

Supplement: Supplementary Figure 2 — Selection of prognostic key genes and development of a 4-gene risk model using LASSO-Cox regression analysis. (A) LASSO coefficient profiles of the 42 intersecting prognostic key genes identified in the TCGA-LAML and GSE71014 datasets. (B) The selection of tuning parameter (λ) in LASSO-Cox regression analysis was conducted using a robust technique of 10-fold cross-validation. [file Image_2.tif]
